# Supplementary material for: Effect of LDHA Inhibition on TNF-α-Induced Cell Migration in Esophageal Cancers
Source: Int J Mol Sci. 2022 Dec 16;23(24):16062. doi: 10.3390/ijms232416062 (PMC9785069; doi:10.3390/ijms232416062)
Supplement: Supplementary file 1 [file ijms-23-16062-s001.zip › supplementary materials.pdf]

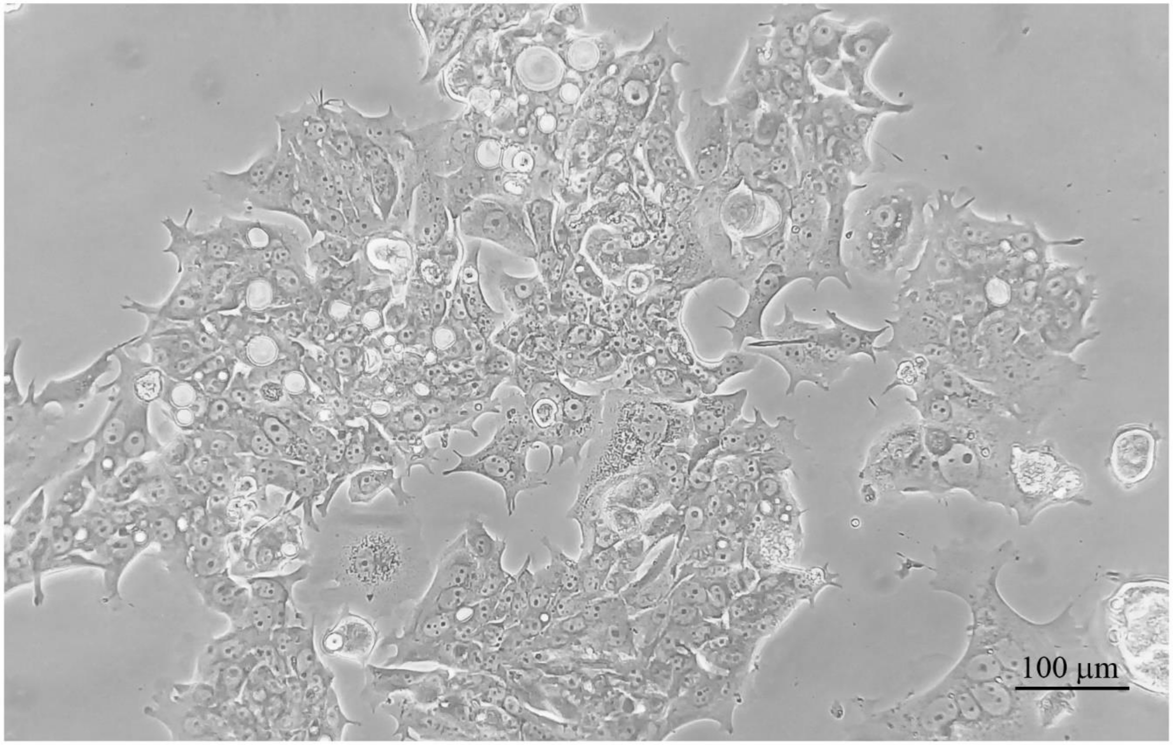

% Match	ATCC cat no	Designation	D5S818	D13S317	D7S820	D16S539	vWA	TH01	AMEL	TPOX	CSF1PO	Origin
100	STRB3221	ESCC-7	12	12,15	10,11	11,12	14,16	9,10	X,Y	8	10,11	European, East

Figure S1. Morphology and DNA fingerprinting of EC7 cells. A light micrograph of the newly established EC7 esophageal cancer cell line and their STR profile obtained by STR analysis according to the ATCC world standard (2020).
